# Glucagon induces translocation of glucokinase from the cytoplasm to the nucleus of hepatocytes by transfer between 6-phosphofructo 2-kinase/fructose 2,6-bisphosphatase-2 and the glucokinase regulatory protein

**DOI:** 10.1016/j.bbamcr.2014.02.006

**Published:** 2014-06

**Authors:** Kirsty S. Cullen, Ziad H. Al-Oanzi, Finbarr P.M. O'Harte, Loranne Agius, Catherine Arden

**Affiliations:** a Institute of Cellular Medicine, Newcastle University, Newcastle Upon Tyne, UK; b Department of Laboratory Medicine, Al-Jouf University, Sakaka, Saudi Arabia; c The Saad Centre for Pharmacy & Diabetes, School of Biomedical Sciences, University of Ulster, Coleraine, UK

**Keywords:** BiFC, bimolecular fluorescence complementation, cAMP, cyclic adenosine monophosphate, EPAC, exchange protein directly activated by cAMP, F26P_2_, fructose 2,6-bisphosphate, GAPDH, glyceraldehyde 3-phosphate dehydrogenase, GK, glucokinase, GKRP, glucokinase regulatory protein, G6pc, glucose 6-phosphatase, mRFP, red fluorescent protein, N/C ratio, nuclear-to-cytoplasmic ratio, PepO, desHis^1^Pro^4^Glu^9^-glucagon, PepR, desHis^1^Pro^4^Glu^9^Lys^12^(γ-glutamyl PAL)glucagon-amide, Phos-a, glycogen phosphorylase, PFK2/FBPase2, 6-phosphofructo 2-kinase/fructose 2,6-bisphosphatase-2, PKA, protein kinase A, PLA, proximity ligation assay, YFP, yellow fluorescent protein, Glucokinase, Glucokinase regulatory protein, 6-phosphofructo 2-kinase/fructose 2,6 bisphosphatase-2, Hepatocyte, Glucagon

## Abstract

Glucokinase activity is a major determinant of hepatic glucose metabolism and blood glucose homeostasis. Liver glucokinase activity is regulated acutely by adaptive translocation between the nucleus and the cytoplasm through binding and dissociation from its regulatory protein (GKRP) in the nucleus. Whilst the effect of glucose on this mechanism is well established, the role of hormones in regulating glucokinase location and its interaction with binding proteins remains unsettled. Here we show that treatment of rat hepatocytes with 25 mM glucose caused decreased binding of glucokinase to GKRP, translocation from the nucleus and increased binding to 6-phosphofructo 2-kinase/fructose 2,6 bisphosphatase-2 (PFK2/FBPase2) in the cytoplasm. Glucagon caused dissociation of glucokinase from PFK2/FBPase2, concomitant with phosphorylation of PFK2/FBPase2 on Ser-32, uptake of glucokinase into the nucleus and increased interaction with GKRP. Two novel glucagon receptor antagonists attenuated the action of glucagon. This establishes an unequivocal role for hormonal control of glucokinase translocation. Given that glucagon excess contributes to the pathogenesis of diabetes, glucagon may play a role in the defect in glucokinase translocation and activity evident in animal models and human diabetes.

## Highlights

• Hepatic glucokinase activity is acutely regulated by its cellular location.
• High glucose translocates glucokinase from nuclear GKRP to cytoplasmic PFK2/FBPase2.
• Here we show that glucagon counteracts glucokinase translocation by elevated glucose.
• This effect of glucagon is reversed by novel glucagon receptor antagonists.
• This study supports a role for glucagon in regulating glucokinase translocation.

## 1 Introduction

The liver plays a vital role in blood glucose homeostasis by production of glucose in the fasted state and efficient removal of glucose in the post-prandial state in response to portal hyperglycaemia for storage of glucose as glycogen or conversion to triacylglycerol [1]. Central to this process is the responsiveness of the liver to the hormone glucagon [1–3]. In normal physiology, glucagon is elevated in the post-absorptive state and acts on the liver to stimulate glucose production *via* glycogenolysis and gluconeogenesis to maintain blood glucose homeostasis [1,2,4]. After a carbohydrate-containing meal, the elevation in insulin suppresses glucagon secretion and thereby hepatic glucose production. However, in type 2 diabetes the deficiency in insulin secretion results in post-prandial hyperglucagonaemic and inadequate suppression of glucose production [2,3]. Whilst the mechanisms involved in the regulation of glycogenolysis and gluconeogenesis by glucagon have been well characterised, the effects of glucagon excess on glucose utilisation have not been fully elucidated.

Glucose metabolism by the liver is critically dependent on the activity of glucokinase, which catalyses the first-step in glucose metabolism [5,6]. Two major mechanisms are involved in the regulation of glucokinase activity: transcriptional mechanisms which account for chronic changes in protein expression [6,7] and translocation from the nucleus to the cytoplasm in response to portal hyperglycaemia or low concentrations of fructose, which accounts for the acute changes in postprandial glucose disposal [5,8].

Sequestration of glucokinase in the hepatocyte nucleus at basal glucose concentrations is regulated by binding to its inhibitory protein (GKRP) [8–10]. Stimulation with elevated concentrations of glucose (> 10 mM) or micromolar concentrations of fructose or other precursors of fructose 1-phosphate causes the dissociation of the glucokinase–GKRP complex, allowing translocation of glucokinase from the nucleus to the cytoplasm, with consequent activation and stimulation of glycogen synthesis [5,6,8]. Various lines of evidence implicate a potential role for phosphofructo 2-kinase/fructose 2,6-bisphosphate (PFK2/FBPase2) as a cytoplasmic binding partner of glucokinase [11,12]. Whilst a role for glucose in regulating glucose translocation is well established [5,6], the role of hormones on glucokinase translocation and its interaction with PFK2/FBPase2 remains unsettled [6,13]. Compelling evidence for an over-riding role for glucagon excess in the pathogenesis of diabetes [3,14] and development of non-invasive methods for estimating glucokinase activity in man based on the assumption that glucokinase activity responds to glucose but not to hormones [15,16], calls for a critical re-evaluation of the effect of glucagon on glucokinase translocation and activity. The aims of this study were to investigate whether glucagon acutely regulates glucokinase translocation and binding to its binding partners GKRP and PFK2/FBPase2.

## 2 Materials and methods

### 2.1 Reagents

Proximity ligation assay reagents were from Olink (Uppsala, Sweden); cAMP lysis buffer was from GE healthcare (Buckinghamshire, UK); cAMP HTRF femto 2 kit was from Cisbio Bioassays (Codolet, France); Synergi C-12 column was from Phenomonex (Cheshire, UK); Site-directed mutagenesis kit was from Agilent Technologies (Berkshire, UK); NE-PER nuclear extraction kit was from Thermo Scientific (Rockford, IL); beta-actin antibody was from Sigma-Aldrich (Poole, UK); Glucokinase antibody was from Santa Cruz Biotechnology (Santa Cruz, CA); GAPDH antibody was from Hytest (Turku, Finland); Lamin A/C antibody was from New England Biolabs (Hitchin, UK); GKRP antibody was from Santa Cruz Biotechnology (Santa Cruz, CA); Total PFK2/FBPase2 antibody was from Simone Baltrusch (University of Rostock, Germany); PFK2/FBPase2 vector was from Alex Lange (University of Minnesota, Minneapolis, MN); Yellow fluorescent protein (YFP)-fragment vectors were from Tom Kerppola (University of Michigan, Ann Arbor, MI).

### 2.2 Hepatocyte isolation/cell culture

Hepatocytes were isolated from male Wistar rats fed *ad libitum* [12] obtained from Harlan (Bicester, UK). Procedures conformed to Home Office regulations and were approved by the local ethics committee. COS1 cells were cultured as in [17]. Cells were seeded on gelatine-coated coverslips for immunostaining, glass chambers for live-cell imaging or 24-well plates for enzyme activity, metabolite determination and flux analysis. Hepatocytes were cultured overnight in MEM in the presence of 10 nM dexamethasone and 10 nM insulin prior to treatment. All treatments were performed in the absence of dexamethasone and insulin.

### 2.3 Immunostaining

Hepatocyte monolayers were fixed with 4% paraformaldehyde in PBS and immunostained for glucokinase or PFK2/FBPase2 as in [12]. Nuclei were counterstained using Hoechst 33342. Cells were imaged for AlexaFluor-488 at excitation 465–495 nm; emission 515–555 nm and Hoechst at excitation 330–380 nm; emission 420 nm using a Nikon E400 microscope (× 60). For method validation, 30 fields from 3 coverslips were imaged (700–950 cells). For subsequent measurements, 10 fields from 2 coverslips were imaged (80–100 cells). Fields were selected at random based on Hoechst staining. The mean pixel intensity for the nucleus and cytoplasm was analysed using Image ProPlus Software and the nuclear-to-cytoplasmic (N/C) ratio calculated for each individual cell.

### 2.4 Glucagon antagonists

Novel glucagon receptor antagonists desHis^1^Pro^4^Glu^9^glucagon-amide (PepO) and the acylated peptide desHis^1^Pro^4^Glu^9^Lys^12^(γ-glutamyl PAL)glucagon-amide (PepR) were produced by Fmoc solid phase peptide synthesis by GL Biochem Ltd. (Shanghai, China). All peptides were > 95% pure as determined by reversed-phase HPLC analysis using acetonitrile gradient elution on a Synergi C-12 column (250 × 4.6 mm). Molecular masses were checked by MALDI-TOF mass spectrometry using a Voyager-DE Biospectrometry Workstation (PerSeptive Biosytems, Framingham, MA, USA).

### 2.5 Proximity ligation assay (PLA)

Hepatocyte monolayers were fixed with 4% paraformaldehyde in PBS and PLA performed as in [18] using antibodies against either glucokinase and GKRP or glucokinase and PFK2/FBPase2. 20 fields from 2 to 3 coverslips/condition were selected at random based on Hoechst staining. The interaction was quantified using Blobfinder software based on either the total number of dots in the nuclear or cytoplasmic compartments or as total intensity of dots in the cell.

### 2.6 Enzyme activity, metabolite determination and glucose flux

Free, bound and total glucokinase activities were determined as in [12]. Free and bound glucokinase activities are expressed as percentage of total activity and total glucokinase activity expressed as mu/mg protein. Glucose phosphorylation and glycolysis were determined as in [12] and lactate production as in [19] and are expressed as nmol/h/mg protein. Glycogen phosphorylase activity was determined as in [20] and is expressed as mU/mg protein. For cAMP determination, cells were lysed using cAMP lysis buffer and cAMP concentrations quantified using the femto 2 HTRF kit as outlined by the manufacturer's instructions. cAMP concentrations are expressed as nmol/mg protein. Fructose 2,6-bisphosphate levels were determined as in [12] and are expressed as fold change relative to the absence of glucagon.

### 2.7 mRNA determination

Glucose 6-phosphatase (G6pc) mRNA levels were determined as described in [7]. Relative mRNA levels were calculated by the Δ cycle threshold method and were normalised to cyclophilin mRNA levels. Results were expressed relative to 5 mM glucose.

### 2.8 Bimolecular fluorescence complementation (BiFC)

Glucokinase-YN155, PFK2/FBPase2-YC155 and mRFP constructs were generated and the BiFC assay performed as in [17]. The serine 32 residue on the PFK2 domain was mutated to alanine (TCC to GCC) or aspartate (TCC to GAC) using site-directed mutagenesis (S32A-fwd: CGG CGA AGG GGC GCC TCC ATA CCA C; S32A-rev: GTG GTA TGG AGG CGC CCC TTC GCC G; S32D-fwd: GCA ACG GCG AAG GGG CGA CTC CAT ACC ACA GTT C; S32D-rev: GAA CTG TGG TAT GGA GTC GCC CCT TCG CCG TGG C). Sequences were confirmed by DNA sequencing and protein translation by western blotting. Constructs were co-expressed in COS1 cells and live-cell fluorescence visualised using a Nikon TE2000 fluorescence microscope (× 100). Cells were imaged for yellow fluorescent protein (YFP, Excitation 500/20 nm; Emission 535/30 nm), red fluorescent protein (mRFP, Excitation 575/25 nm; Emission 632/40 nm) and Hoechst (Excitation 402/15 nm; Emission 455/20 nm). For quantification, the number of cells expressing mRFP (transfection control) and YFP (BiFC signal) was calculated (20-fields) and YFP-positive cells expressed as a percentage of mRFP-positive cells.

### 2.9 Nuclear fractionation

Hepatocytes were fractionated into nuclear and cytoplasmic compartments using either the NE-PER nuclear extraction kit or as in [21]. An aliquot (30 μg) of cytoplasmic or nuclear protein was subjected to SDS-PAGE following by western blotting.

### 2.10 Western blotting

Immunoreactivity towards PFK2-(P) [12], GAPDH, Lamin A/C, total PFK2 and β-actin was determined by SDS-PAGE using a 10% polyacrylamide gel and immunoblotting and was quantified using densitometry.

### 2.11 Statistical analysis

Results are expressed as means ± s.e.m. for the number of cell preparations and values compared using either the unpaired Student's *t*-test or by one-way ANOVA followed by Bonferroni's test.

## 3 Results

### 3.1 The nuclear-to-cytoplasmic ratio is a more sensitive index of glucokinase translocation than nuclear intensity

**Fig. 1:**
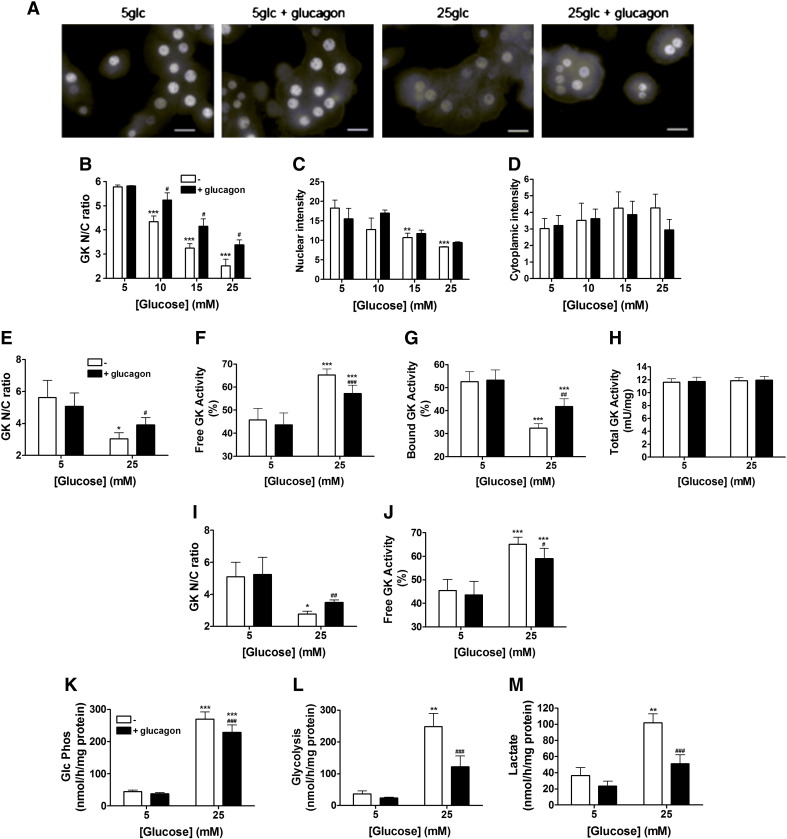
Glucagon inhibits glucose-induced glucokinase translocation and glucose phosphorylation. (A) Hepatocytes were incubated at either 5 or 25 mM glucose without or with 100 nM glucagon for 1 h and glucokinase localization determined using immunostaining. Between 705 and 921 nuclei were imaged for each condition. Images are representative of randomly selected fields from 8 experiments. Scale bars: 20 μm. (B–D) Hepatocytes were incubated for 30 min at 5, 10, 15 or 25 mM glucose without or with 100 nM glucagon for determination of glucokinase N/C ratio (B), nuclear intensity (C) and cytoplasmic intensity (D). (E–H) Hepatocytes were incubated for 30 min at 5 *vs*. 25 mM glucose without or with 100 nM glucagon for determination of glucokinase N/C ratio (E), free glucokinase activity (F), bound glucokinase activity (G) or total glucokinase activity (H). (I–J) Hepatocytes were incubated for 1 h at 5 *vs*. 25 mM glucose followed by addition of 100 nM glucagon for a further 30 min for determination of N/C ratio (I) or free glucokinase activity (J). (K–M) Hepatocytes were incubated at 5 *vs*. 25 mM glucose with or without 100 nM glucagon for 1 h for determination of glucose phosphorylation (K), glycolysis (L) or lactate production (M). Means ± s.e.m. of 3–6 independent experiments. *p < 0.05, **p < 0.01, ***p < 0.005 effect of glucose; ^#^p < 0.05, ^##^p < 0.01, ^###^p < 0.005 effect of glucagon.

Whereas the effect of elevated glucose (25 mM *vs*. 5 mM) in translocating glucokinase from the nucleus to the cytoplasm in hepatocytes is well established [12,13,22–24], the effect of glucagon remains contentious [6,13,23,25] and an exclusive role for glucose as distinct from hormone action is commonly inferred [15]. Possible explanations for the discordance in hormonal control include differences in glucose concentration [6,13,22,25] or whether data is analysed as the nuclear to cytoplasmic (N/C) ratio calculated per cell [23,24] rather than the average nuclear or cytoplasmic intensities [13,26]. To test for these possibilities, we determined the effects of glucagon on glucokinase translocation at 5 mM or 25 mM glucose and analysed the data from N/C ratios and also from nuclear or cytoplasmic intensities from > 700 cells per condition (Table 1). High glucose increased cytoplasmic intensity by 33% (7.9 ± 3.3 to 10.5 ± 3.7) and decreased both nuclear intensity and N/C ratio by 30% and 48%, respectively (nuclear 62.2 ± 27.0 to 43.8 ± 14.4; N/C ratio 8.4 ± 3.3 to 4.4 ± 1.4) (Fig. 1A, Table 1). Glucagon had no significant effect on glucokinase localization at 5 mM glucose but attenuated the effects of 25 mM glucose, quantified as a 13% decrease in cytoplasmic intensity (10.5 ± 3.7 to 9.1 ± 3.4), an 11% increase in nuclear intensity (43.8 ± 14.4 to 48.8 ± 17.7) and a 32% increase in the N/C ratio (4.4 ± 1.4 to 5.8 ± 2.3) (Fig. 1A, Table 1). Fractional changes in response to 25 mM glucose or glucagon were greater for the N/C ratio than for nuclear or cytoplasmic intensities (Table 1) and accordingly the power calculations showed that a larger sample size is required to detect significant differences in translocation from nuclear or cytoplasmic intensities (nuclear: 234; cytoplasmic: 178) as opposed to N/C ratio (N/C ratio: 60) (Table 1). Based on these power calculations, imaging of 80–100 nuclei/condition was necessary to detect significant glucokinase translocation using the N/C ratio.

We next tested the effect of glucose concentration on the glucagon response. Glucokinase translocation determined from the N/C ratio increased significantly and progressively at glucose concentrations of 10 mM to 25 mM glucose relative to 5 mM (Fig. 1B). Glucagon partially attenuated the effects of elevated glucose (10 to 25 mM) but had no effect on glucokinase translocation at 5 mM glucose (Fig. 1B). Analysis of this data based on individual nuclear or cytoplasmic intensities showed greater variability compared to the N/C ratio (CV values: Nuclear = 5–23%, Cytoplasmic = 16–30%; N/C = 2–13%) (Fig. 1B–D) and the changes in glucokinase translocation in response to glucagon were not significant (Fig. 1C–D), in agreement with previous findings [13]. For the rest of the study, glucokinase translocation was determined using the N/C ratio, which offers a sensitive semi-quantitative method for the measurement of glucokinase translocation.

### 3.2 Glucagon alters both glucokinase translocation and activity

To further confirm the effect of glucagon on glucokinase translocation, we measured free cytoplasmic glucokinase activity released from digitonin-permeabilised hepatocytes [27] and the rate of detritiation of [2-^3^H]glucose which is an approximate measure of glucose phosphorylation [28]. Stimulation with high glucose caused an increase in free glucokinase activity (Fig. 1F) and a decrease in bound glucokinase activity (Fig. 1G), representing translocation of glucokinase from the nucleus to the cytoplasm. The glucose-induced changes in free and bound glucokinase activity were significantly attenuated by glucagon (Fig. 1F, G) consistent with the changes in N/C ratio (Fig. 1E). Due to the semi-quantitative nature of the immunostaining technique, total glucokinase concentration could not be accurately determined using absolute nuclear plus cytoplasmic intensity levels. Therefore, the effect of glucagon on cellular glucokinase content was assessed using total glucokinase activity. There was no change in total glucokinase activity, suggesting that acute glucagon treatment alters glucokinase distribution rather than activity/expression (Fig. 1H).

**Fig. 2:**
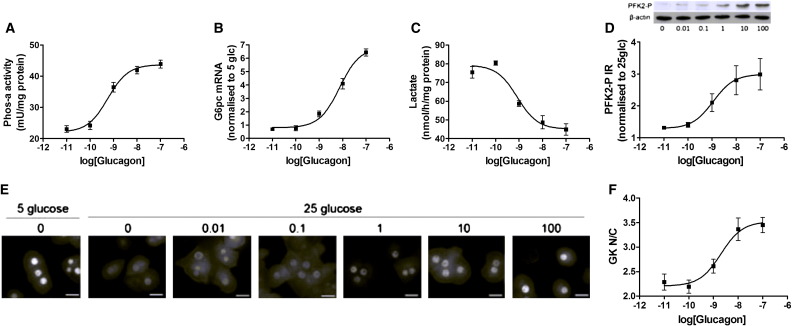
Glucokinase translocation has a similar affinity to glucagon as established metabolic effects. Hepatocytes were incubated at 5 (B) or 25 mM (A, C–F) glucose with increasing concentrations of glucagon (0.01–100 nM) for (A) 5 min for glycogen phosphorylase activity, (B) 2 h for G6pc mRNA levels, (C) 30 min for lactate concentration, (D) 10 min for phosphorylation of PFK2/FBPase2 at Ser-32, and (E–F) 30 min for glucokinase N/C ratio. Data are plotted against glucagon concentration on a log plot and the EC_50_ for glucagon calculated by non-linear regression analysis. Means ± s.e.m. of n = 4 from 2 independent experiments. Scale bars: 20 μm.

Inhibition of glucokinase translocation by glucagon was also observed when hepatocytes were pre-equilibrated with 25 mM glucose for 30 min to allow maximum translocation prior to glucagon challenge (Fig. 1I, J). The effects of glucagon on glucokinase translocation at 25 mM glucose were associated with attenuation of metabolism of [2-^3^H]glucose (Fig. 2K) and glycolysis, determined either radiochemically or from lactate formation (Fig. 2L, M). Fractional inhibition of metabolism of [2-^3^H]glucose was smaller than inhibition of glycolysis (19% *vs*. 61–62%), consistent with additional effects of glucagon downstream of glucose phosphorylation [29].

### 3.3 The affinity for glucagon is similar for glucokinase translocation as for established effects of the hormone

We next compared the effects of glucagon concentration on glucokinase translocation with three established cellular responses to the hormone: activation of glycogen phosphorylase, phosphorylation of PFK2/FBPase2 at Ser-32 which determines the cellular concentration of fructose 2, 6-bisphosphate (F26P_2_) and thereby flux through glycolysis, and mRNA expression of glucose 6-phosphatase (G6pc) [30–33]. Stimulation of hepatocytes with 1–100 nM glucagon caused an increase in glycogen phosphorylase activity, G6pc mRNA levels, phosphorylation of PFK2/FBPase2 at Ser-32 and a decrease in glycolysis as determined by lactate concentration (Fig. 2A–D), and an increase in the glucokinase N/C ratio (Fig. 2E, F). All five parameters showed similar affinity for glucagon with an EC_50_ between 1 and 10 nM (EC_50_ values: Phos-a = 0.57 ± 0.14; G6pc = 7.43 ± 0.11; PFK2-P = 1.12 ± 0.40; Lactate = 0.84 ± 0.19 nM; Glucokinase N/C ratio = 2.04 ± 0.29).

### 3.4 Glucagon antagonists block glucagon-induced glucokinase movement

**Fig. 3:**
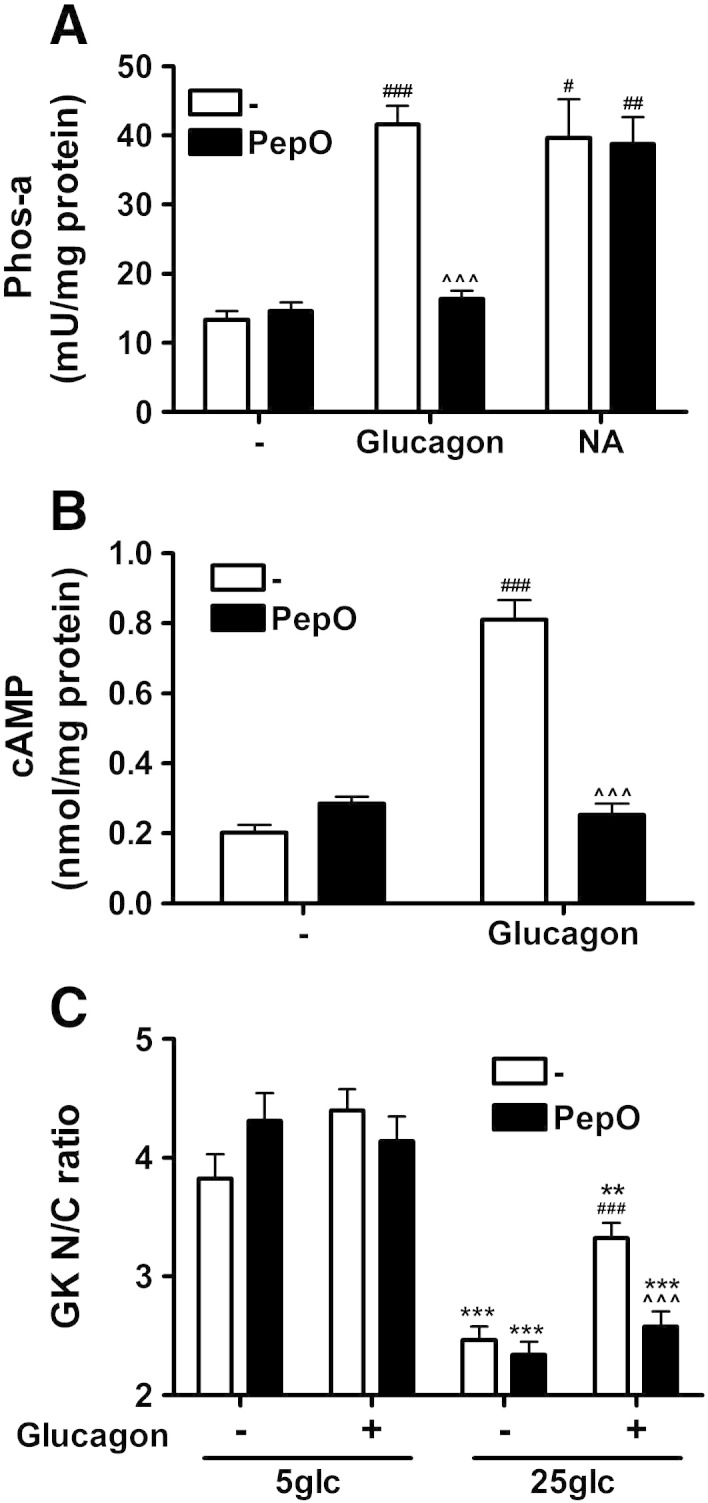
A novel glucagon antagonist inhibits glucagon-induced glucokinase translocation. (A) Hepatocytes were incubated for 1 min without or with 10 μM desHis^1^Pro^4^Glu^9^-glucagon (PepO) prior to the addition of 10 nM glucagon or 100 μM noradrenaline (NA) for 30 min for the determination of glycogen phosphorylase activity. (B) Hepatocytes were incubated for 1 min without or with 10 μM PepO prior to the addition of 10 nM glucagon for 30 min for the determination of cAMP levels. (C) Hepatocytes were incubated for 1 min without or with 10 μM PepO prior to the addition of 10 nM glucagon for 30 min at 5 *vs*. 25 mM glucose for the determination of glucokinase N/C ratio. Means ± s.e.m. of n = 6–8 from 3 independent experiments. **p < 0.01, ***p < 0.005 effect of glucose; ^#^p < 0.05, ^##^p < 0.01, ^###^p < 0.005 effect of glucagon or noradrenaline; ^^^p < 0.005 effect of glucagon antagonist.

We next confirmed the specificity of the glucagon effect on glucokinase translocation with novel glucagon receptor antagonists [34]. Pre-incubation of hepatocytes with the novel antagonist desHis^1^Pro^4^Glu^9^-glucagon (PepO) inhibited the activation of glycogen phosphorylase by glucagon but not that by noradrenaline (Fig. 3A), confirming its specificity. PepO inhibited the glucagon-induced increase in cellular cAMP and the translocation of glucokinase to the nucleus at elevated glucose (Fig. 3B, C). Similar results were obtained for the acylated analogue desHis^1^Pro^4^Glu^9^Lys^12^(γ-glutamyl PAL)glucagon-amide (results not shown). These results confirm the involvement of glucagon signalling in glucokinase translocation.

### 3.5 Glucagon regulates glucokinase translocation *via* a cAMP/PKA dependent mechanism

**Fig. 4:**
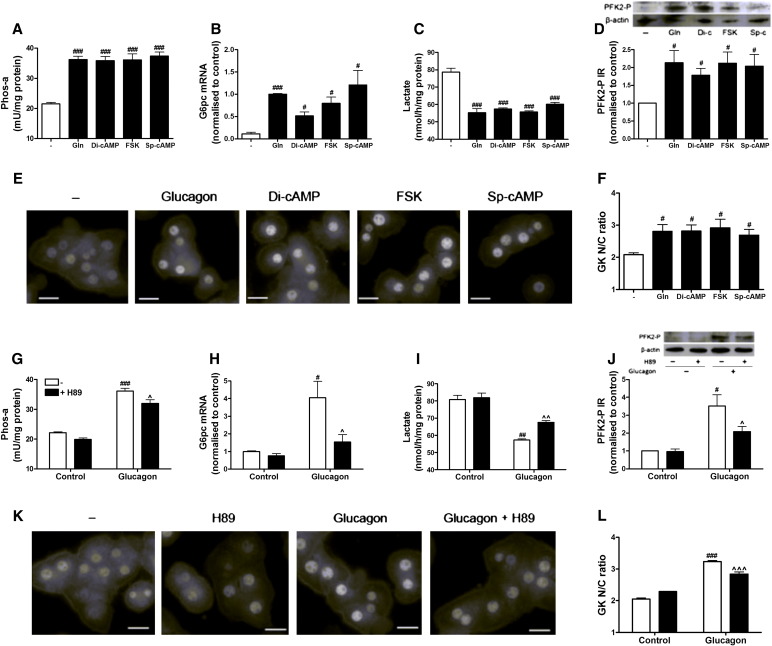
Glucagon regulates glucokinase translocation *via* a protein kinase A dependent mechanism. (A–F). Hepatocytes were incubated at 5 (B) or 25 mM glucose (A, C–F) with or without 10 nM glucagon, 100 μM dibutyryl cAMP (Di-cAMP), 20 μM forskolin (FSK) or 100 μM Sp-cAMP for (A) 5 min for glycogen phosphorylase activity, (B) 2 h for G6pc mRNA levels, (C) 30 min for lactate concentration, (D) 10 min for phosphorylation of PFK2/FBPase2 at Ser-32, and (E–F) 30 min for glucokinase nuclear: cytoplasmic (N/C) ratio. (G–L). Hepatocytes were pre-incubated for 30 min with or without 10 μM H-89. Cells were then stimulated with 5 (H) or 25 mM glucose (G, I–L) with or without 10 nM glucagon for (G) 5 min for glycogen phosphorylase activity, (H) 2 h for G6pc mRNA levels, (I) 30 min for lactate concentration, (J) 10 min for phosphorylation of PFK2/FBPase2 at Ser-32, and (K-L) 30 min for glucokinase nuclear: cytoplasmic (N/C) ratio. Means ± s.e.m. of n = 4 from 2 independent experiments. ^#^p < 0.05, ^##^p < 0.01, ^###^p < 0.005 effect of glucagon or cAMP analogue. ^p < 0.05, ^^p < 0.01, ^^^p < 0.005 effect of H-89. Scale bars: 20 μm.

To determine whether glucagon exerted its effects on glucokinase translocation by a protein kinase A (PKA)-dependent mechanism, hepatocytes were stimulated with PKA-selective or non-selective cAMP analogues. Both selective (Sp-cAMP) and non-selective (forskolin and dibutyryl-cAMP) cAMP analogues mimicked the effect of glucagon on glycogen phosphorylase, G6pc mRNA expression, lactate production, phosphorylation of PFK2/FBPase2 and glucokinase translocation (Fig. 4A–F). Pre-treatment of hepatocytes with the PKA inhibitor H89 partially reversed the effect of glucagon on these parameters (Fig. 4G–L), whilst the exchange protein directly activated by cAMP (EPAC) inhibitor Brefeldin A had no effect (results not shown). These results suggest that glucagon regulates the localization of glucokinase by a cAMP/PKA-dependent mechanism.

### 3.6 Glucagon disrupts the glucokinase–PFK2/FBPase2 complex

**Fig. 5:**
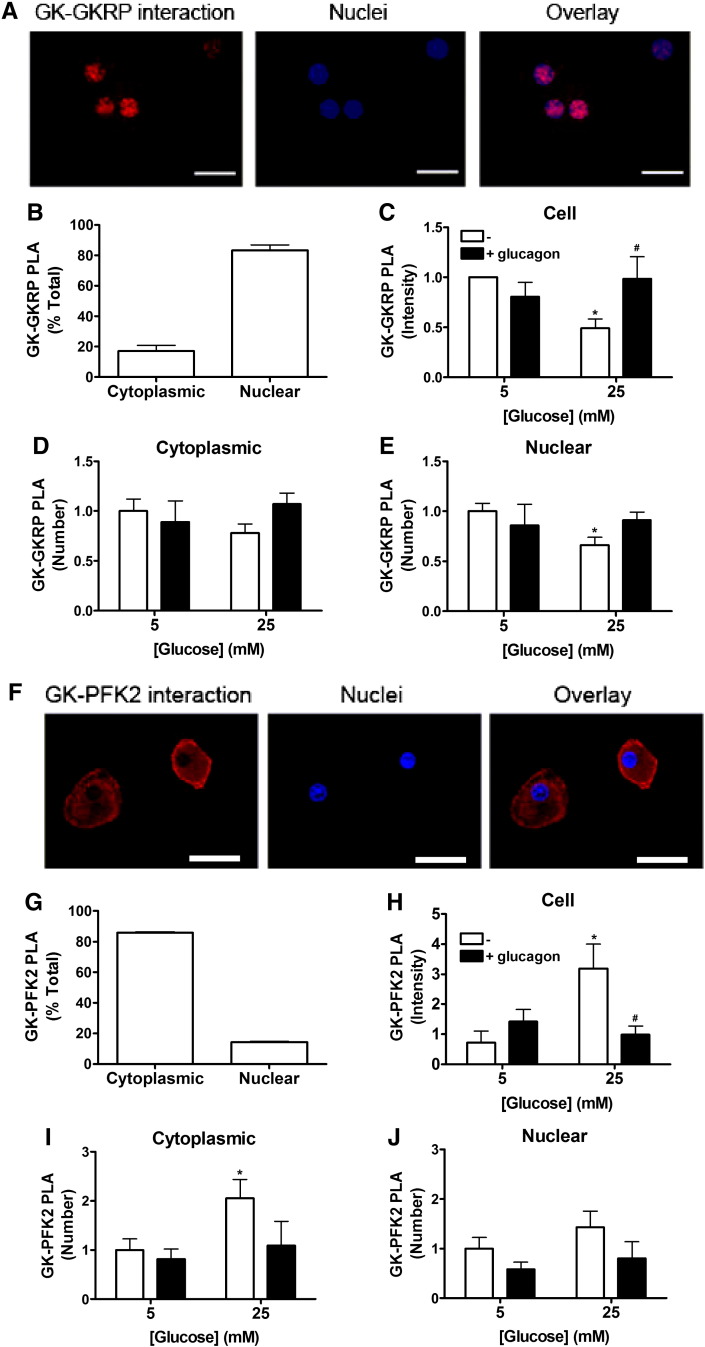
Glucagon disrupts the glucokinase–PFK2/FBPase2 complex. (A–B) Hepatocytes grown on coverslips were incubated at 5 mM glucose for 30 min and the proximity ligation assay (PLA) used to detect the interaction between glucokinase and GKRP (red). Nuclei were stained using Hoechst 33342 (blue). (A) Images are representative of randomly selected fields from 4 experiments. (B) The number of dots in the nuclear and cytoplasmic compartments was counted and results expressed as a percentage of total dots. (C–E) Hepatocytes were incubated at 5 or 25 mM glucose with or without 100 nM glucagon for 30 min for determination of the glucokinase–GKRP interaction using PLA. (C) The total intensity of dots in each cell was quantified. (D) The total number of dots in the cytoplasm of each cell was quantified. (E) The total number of dots in the nucleus of each cell was quantified. Results were normalised to 5 mM glucose. (F–G) Hepatocytes were incubated at 25 mM glucose for 30 min and PLA used to detect the interaction between glucokinase and PFK2/FBPase2 (red). (F) Images are representative of 8 experiments. (G) The number of dots in the nuclear and cytoplasmic compartments was counted and results expressed as a percentage of total dots. (H–J) Hepatocytes were incubated at 5 or 25 mM glucose with or without 100 nM glucagon for 30 min for determination of the glucokinase–PFK2/FBPase2 interaction using PLA. (H) The total intensity of dots in each cell was quantified. (I) The total number of dots in the cytoplasm of each cell was quantified. (J) The total number of dots in the nucleus of each cell was quantified. Results were normalised to 5 mM glucose. Means ± s.e.m. of 3–7 independent experiments. *p < 0.05 effect of glucose. ^#^p < 0.05 effect of glucagon or dibutyryl cAMP. Scale bars: 20 μm (A) or 30 μm (F).

The accumulation of glucokinase in the hepatocyte nucleus is contingent on binding to GKRP as supported by the exclusive cytoplasmic location in GKRP KO models [9,10]. A candidate binding partner in the cytoplasm is PFK2/FBPase2 [11,12]. We used a proximity ligation assay (PLA) to quantify the interactions of glucokinase with GKRP and PFK2/FBPase2. This assay uses primary antibodies raised against two candidate interacting proteins followed by secondary antibodies tagged with complimentary oligonucleotides that can be ligated when in close proximity. The product is then amplified for visualisation [18]. This enables objective quantification of two proximity-linked proteins visualised as red dots (Fig. 5A, F). We first validated the technique from the interaction between glucokinase and GKRP. As expected, at 5 mM glucose the interaction between glucokinase and GKRP was predominately localised to the nuclear compartment (Fig. 5A, B). Stimulation with 25 mM glucose caused a decrease in the glucokinase–GKRP interaction in the total cell compartment and in the cytoplasm or nucleus (Fig. 5C–E). The effect of glucose was reversed by the addition of glucagon (Fig. 5C–E).

In contrast, the glucokinase–PFK2/FBPase2 complex predominately localised to the cytoplasmic compartment (Fig. 5F, G). The staining pattern indicates a peripheral location of the glucokinase–PFK2/FBPase2 interaction close to the plasma membrane, consistent with previous reports of glucokinase localization to this compartment [24]. No signal was detected for isotype negative controls and overexpression of glucokinase and PFK2/FBPase2 increased PLA signal intensity, confirming the specificity of the signal (results not shown). The intensity of amplified products (representing glucokinase–PFK2/FBPase2) was significantly increased at 25 mM compared with 5 mM glucose and this effect was reversed by glucagon (Fig. 5H, J).

**Fig. 6:**
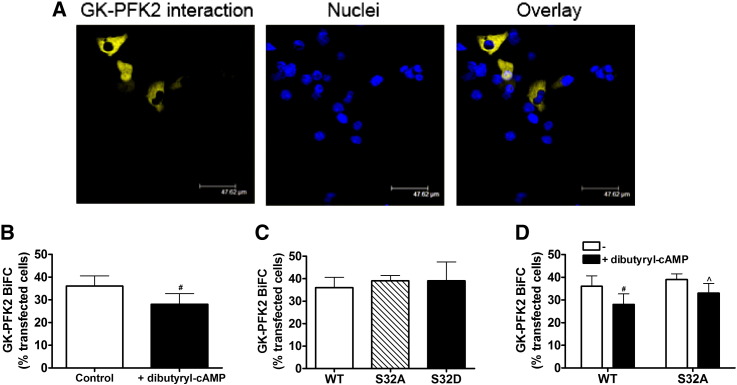
cAMP disrupts the glucokinase–PFK2/FBPase2 complex. Cos 1 cells grown in glass chambers were transfected with 1 μg/well glucokinase-YN155 wild-type PFK2/FBPase2-YC155 (WT), Ser-32-Ala PFK2/FBPase2-YC155 (S32A) or Ser-32-Asp PFK2/FBPase2-YC155 (S32D) and 0.2 μg/well full-length mRFP protein for 24 h. Cells were then incubated at 25 mM glucose with or without 50 μM dibutyryl cAMP for 1 h for determination of the glucokinase–PFK2/FBPase2 interaction (yellow) using the BiFC assay. Means ± s.e.m. of 3–7 independent experiments. ^#^p < 0.05 effect of dibutyryl cAMP. ^p < 0.05 effect of mutant. Scale bars: 50 μm.

To further confirm the association between glucokinase and PFK2/FBPase2, we used the bimolecular fluorescence complementation (BiFC) assay in heterologous cells. This involves expression of the two proteins of interest as fusion proteins with either the N-terminal or C-terminal halves of YFP. If the two proteins associate, the non-fluorescent N-terminal and C-terminal YFP fragments form a fluorescent complex [17]. COS1 cells transfected with glucokinase-YN155 and PFK2/FBPase2-YC155 demonstrated formation of a BiFC complex (Fig. 6A), confirming an interaction between glucokinase and PFK2/FBPase2 in the cytoplasmic compartment. Formation of this complex was slightly attenuated by dibutyryl cAMP (Fig. 6B). To determine whether phosphorylation of PFK2/FBPase2 at Ser-32 [29,31] is involved in regulating the glucokinase–PFK2/FBPase2 interaction, Ser-32 was mutated to alanine (S32A) or aspartate (S32D). Wild-type and mutant vectors (PFK2/FBPase2-YC155) were co-expressed with glucokinase-YN155 in COS1 cells for BiFC analysis of the glucokinase–PFK2/FBPase2 interaction. The S32A and S32D mutants formed a complex with glucokinase to the same extent as wild-type (Fig. 6C). Dibutyryl cAMP significantly attenuated complex formation for wild-type enzyme but had no significant effect for the S32A mutant (Fig. 6D). These results support involvement of Ser-32 phosphorylation in complex formation with glucokinase.

### 3.7 Glucagon alters the subcellular location of PFK2/FBPase2 in hepatocytes

**Fig. 7:**
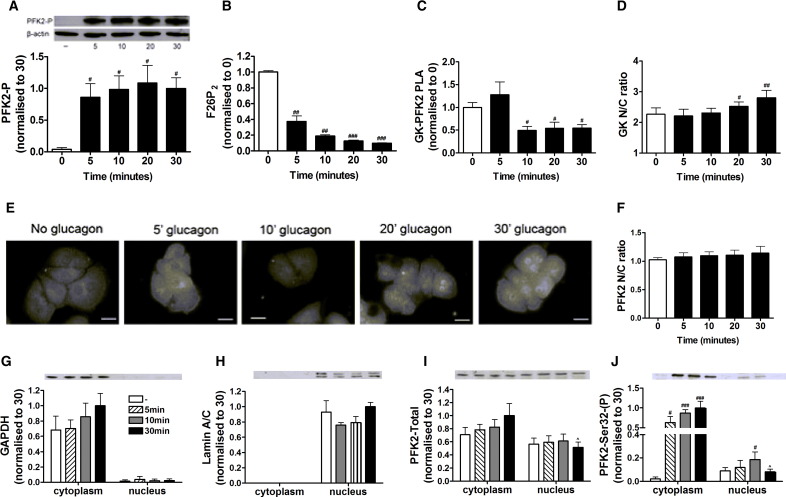
Glucagon alters the subcellular location of PFK2/FBPase2 in hepatocytes. (A–F) Hepatocytes were incubated with 25 mM glucose with or without 100 nM glucagon for 5, 10, 20 or 30 min for the determination of phosphorylation of PFK2/FBPase2 at Ser-32 by western blotting (A), fructose 2,6-bisphosphate (F26P_2_) concentration (B), the glucokinase–PFK2/FBPase2 interaction using PLA (C), glucokinase N/C ratio (D), PFK2/FBPase2 localization (E) and the PFK2/FBPase2 N/C ratio (F). (G–J) Hepatocytes were incubated with 25 mM glucose with or without 100 nM glucagon for 5, 10 or 30 min. The subcellular locations of GAPDH (G), Lamin A/C (H), total PFK2/FBPase2 (I) and PFK2-Ser32(P) (J) were determined by western blotting of nuclear and cytoplasmic fractions. Full size images of the western blots are shown in Supplementary Fig. 1. Means ± s.e.m. of 4–7 independent experiments. ^#^p < 0.05, ^##^p < 0.01, ^###^p < 0.005 effect of glucagon; ^p < 0.05 relative to 20 min time point. Scale bars: 20 μm.

We next determined the time course of glucagon on glucokinase localization and its interaction with PFK2/FBPase2. Glucagon caused phosphorylation of PFK2/FBPase2 at Ser-32 within 5 min (Fig. 7A), it depleted its product fructose 2,6-bisphosphate during 5 to 20 min (Fig. 7B) and dissociated the glucokinase–PFK2/FBPase2 complex at 10 min (Fig. 7C). A significant increase in translocation of glucokinase to the nucleus was detectable after 20 to 30 min (Fig. 7D).

To determine the role of the glucokinase–PFK2/FBPase2 interaction in the glucagon-induced uptake of glucokinase to the nucleus, the location of PFK2/FBPase2 was determined. Previous studies have shown that the ubiquitous isoform of PFK2/FBPase2 (PFKFB3) localises to the nuclear compartment of cancer cells [35] but the location of the liver isoform of PFK2/FBPase2 (PFKFB1) has not been investigated. By immunostaining, the liver isoform of PFK2/FBPase2 can be detected in both the cytoplasmic and nuclear compartments of hepatocytes (Fig. 7E). However, the nuclear sequestration was much less pronounced than for glucokinase (Fig. 1A), in that there was no predominance of PFK2/FBPase2 in the nucleus relative to the cytoplasm in any of the incubation conditions. This and the large degree of heterogeneity in PFK2/FBPase2 localization prevented reliable quantification of the N/C ratio (Fig. 7E, F). Therefore, the location of PFK2/FBPase2 was determined by subcellular fractionation and immunoblotting (Fig. 7G–J, Supplementary Fig. 1). The purity of cytoplasmic and nuclear fractions was confirmed using the cytoplasmic marker GAPDH (Fig. 7G) and the nuclear marker Lamin A/C (Fig. 7H). PFK2/FBPase2 protein localised to both nuclear and cytoplasmic compartments (Fig. 7I), consistent with immunostaining (Fig. 7E). After treatment with glucagon, phosphorylated PFK2/FBPase2 at Ser-32 was detected in both the cytoplasmic and nuclear fractions (Fig. 7J). Nuclear immunoreactivity to both phosphorylated PFK2/FBPase2 (Fig. 7J) and total PFK2/FBPase2 (Fig. 7I) declined at 30 min. This demonstrates that the liver isoform of PFK2/FBPase2 was present in the nucleus and it implicates movement of PFK2/FBPase2 (phosphorylated form) between compartments in response to glucagon.

To determine the role of the glucokinase–PFK2/FBPase2 interaction in the glucagon-induced uptake of glucokinase to the nucleus, the location of PFK2/FBPase2 was determined. Previous studies have shown that the ubiquitous isoform of PFK2/FBPase2 (PFKFB3) localises to the nuclear compartment of cancer cells [35] but the location of the liver isoform of PFK2/FBPase2 (PFKFB1) has not been investigated. By immunostaining, the liver isoform of PFK2/FBPase2 can be detected in both the cytoplasmic and nuclear compartments of hepatocytes (Fig. 7E). However, the nuclear sequestration was much less pronounced than for glucokinase (Fig. 1A), in that there was no predominance of PFK2/FBPase2 in the nucleus relative to the cytoplasm in any of the incubation conditions. This and the large degree of heterogeneity in PFK2/FBPase2 localization prevented reliable quantification of the N/C ratio (Fig. 7E, F). Therefore, the location of PFK2/FBPase2 was determined by subcellular fractionation and immunoblotting (Fig. 7G–J, Supplementary Fig. 1). The purity of cytoplasmic and nuclear fractions was confirmed using the cytoplasmic marker GAPDH (Fig. 7G) and the nuclear marker Lamin A/C (Fig. 7H). PFK2/FBPase2 protein localised to both nuclear and cytoplasmic compartments (Fig. 7I), consistent with immunostaining (Fig. 7E). After treatment with glucagon, phosphorylated PFK2/FBPase2 at Ser-32 was detected in both the cytoplasmic and nuclear fractions (Fig. 7J). Nuclear immunoreactivity to both phosphorylated PFK2/FBPase2 (Fig. 7J) and total PFK2/FBPase2 (Fig. 7I) declined at 30 min. This demonstrates that the liver isoform of PFK2/FBPase2 was present in the nucleus and it implicates movement of PFK2/FBPase2 (phosphorylated form) between compartments in response to glucagon.

## 4 Discussion

The importance of glucokinase translocation in determining the rate of hepatic glucose disposal [5] and also the effect of glucose concentration on glucokinase translocation are well established from studies *in vitro* or *in vivo* from several independent laboratories [13,22–24,26,36–38]. However, the question whether this translocation mechanism is regulated by hormones and specifically by glucagon has been less widely investigated and remains contentious [6,13,22,25]. This question is timely in view of the recognised role of hyperglucagonaemic in human diabetes [3], the potential therapeutic benefit of glucagon antagonists for glycaemic control in diabetes [39], and current methods for assessment of glucokinase activity based on the assumption of exclusive control by glucose as opposed to hormones [15,16].

A key limiting factor in the semi-quantitative analysis of changes in the nuclear-to-cytoplasmic distribution of glucokinase is the large intercellular heterogeneity of expression of glucokinase in both isolated hepatocytes *in vitro* and also in liver *in vivo* [13,23,24,26,36]. This necessitates the analysis of large numbers of cells. To date, two methods have been used for semi-quantitative analysis of glucokinase translocation: either based on the determination of the ratio of cellular nuclear-to-cytoplasmic (N/C) mean pixel intensity [23–25] or from the automated nuclear or cytoplasmic intensity multiplied by the total cytoplasmic or nuclear area estimated with an independent stain [13,26,37]. We show in this study from the analysis of N/C ratio normalised per cell that glucagon antagonises the effect of elevated glucose (10, 15 and 25 mM) on glucokinase translocation but has no significant effect at basal glucose concentration (5 mM). We also confirm that the N/C ratio is a more accurate assessment of translocation than the mean nuclear or cytoplasmic intensity based on cell outline methods consistent with a previous study [24]. The failure to detect the effects of glucagon on glucokinase translocation in recent studies [13] could be explained by the low glucose concentration used, as well as the exclusive analysis of global nuclear or cytoplasmic intensity. We show in this study that the counter-regulatory effect of glucagon on glucose-induced translocation of glucokinase is abolished by glucagon receptor antagonists and mimicked by non-metabolisable cyclic-AMP analogues.

We used the proximity ligation assay (PLA) for the quantification of the protein–protein interaction of glucokinase with either GKRP or PFK2/FBPase2. This assay enables the visualisation of two endogenously expressed proteins that are in close proximity such that the antibody-linked oligonucleotide tag can be ligated [18]. Using this assay we show that at low glucose concentration the interaction between glucokinase and GKRP occurred predominately (though not exclusively) in the nucleus, consistent with the high nuclear to cytoplasmic ratio of GKRP [24,40]. We also show that elevated glucose decreases the glucokinase–GKRP interaction, with concomitant translocation of glucokinase from the nuclear to cytoplasmic compartment and increased binding between glucokinase and PFK2/FBPase2, an interaction that occurs predominately (though not exclusively) in the cytoplasm. Glucokinase has a bilobal structure linked by a flexible hinge [41] and exists as an equilibrium of conformational states ranging from a wide-open state at low glucose concentration to a closed conformation at high glucose [42,43]. Glucokinase binds to GKRP in the wide-open state [44,45] and it binds to PFK2/FBPase2 most likely in the closed conformation [46]. The converse effects of elevated glucose on the interaction of glucokinase with GKRP and PFK2/FBPase2 are therefore consistent with the expected binding to these two proteins. Interestingly, glucagon caused complete reversal of the effect of elevated glucose on both the glucokinase–GKRP and glucokinase–PFK2/FBPase2 interactions (Fig. 5) despite causing only partial reversal of the effect of glucose on the distribution of glucokinase between the cytoplasm and nucleus (Fig. 1). It is noteworthy that whilst immunostaining provides a global measure of the distribution of glucokinase between the nuclear and cytoplasmic compartments, the PLA assay is a measure of two proteins in close proximity and in an orientation that allows the antibody tags to form a complex. Therefore, the signal detected by the PLA assay most likely represents a subset of the glucokinase molecules. In principle, glucokinase may bind simultaneously to more than one protein. For example, shuttling between the cytoplasm and nucleus may involve interaction with additional shuttling proteins, which may mask the epitopes to PFK2/FBPase2 or GKRP or alternatively force the complex into a more or less favourable orientation for ligation of the complementary tags. The marked effect of glucagon in reversing the effects of elevated glucose on both the glucokinase–PFK2/FBPase2 interaction and the glucokinase–GKRP interaction is compelling evidence for hormonal control of glucokinase shuttling through interactions with these partners.

Glucagon regulates the transition from hepatic glucose utilisation in the absorptive state to glucagon production in the post-absorptive state by acute stimulation of glycogenolysis and inhibition of glycolysis [1]. An important component of this response is the phosphorylation of liver PFK2/FBPase2 at Ser-32 [25,29], which increases the bisphosphatase to kinase ratio of the bifunctional enzyme leading to depletion of fructose 2,6-bisphosphate and inhibition of glycolysis and elevation in gluconeogenesis [25]. Here we show that the phosphorylated form of PFK2/FBPase2 is found in the nucleus as well as the cytoplasm and that glucagon alters the amount of phosphorylated PFK2/FBPase2 in the nucleus. This implicates a potential role for PFK2/FBPase2 in the glucagon-induced changes in the subcellular location of glucokinase. We propose the following model: glucagon *via* cAMP/PKA-dependent signalling alters the glucokinase–PFK2/FBPase2 interaction by a mechanism that may involve phosphorylation of PFK2/FBPase2 at Ser-32. These changes enable movement of glucokinase to the nuclear compartment, either in complex with PFK2/FBPase2 or GKRP or both proteins. The BiFC data supports the existence of a multi-protein complex since the cAMP analogue significantly attenuated but did not block the formation of the glucokinase–PFK2/FBPase2 complex. The BiFC assay is carried out in a heterologous cell system lacking GKRP and thus measures the interaction of glucokinase and PFK2/FBPase2 independently of GKRP, which is essential for translocation and sequestration of glucokinase to the nucleus in hepatocytes [47,48]. The failure of the cAMP analogue to block the interaction of glucokinase with PFK2/FBPase2 in the BiFC assay indicates that phosphorylation does not prevent formation of the complex. One possibility that remains to be tested is that phosphorylation may favour formation of a multi-protein complex with GKRP enabling translocation of glucokinase to the nucleus. Further work testing for a trimer complex is required to investigate this possibility.

Several studies have reported that translocation of glucokinase in response to glucose is impaired in animal models of type 2 diabetes [25,37,38,49,50]. The present evidence that glucagon antagonises the action of elevated glucose on translocation of glucokinase from the nucleus and binding to PFK2/FBPase2 in the cytoplasm adds a new perspective to the acute control of glucokinase activity and shows that absolute or relative glucagon excess as occurs in diabetes [3] is most likely a contributing factor to the defective glucokinase translocation in animal models of diabetes [25,37,38,49,50].

The following are the supplementary data related to this article.

Supplementary Fig. 1 Localisation of PFK2/FBPase2 in hepatocytes. Hepatocytes were incubated with 25 mM glucose with or without 100 nM glucagon for 5, 10 or 30 min. The subcellular locations of GAPDH, Lamin A/C, total PFK2/FBPase2 and PFK2-Ser32(P) were determined by western blotting of nuclear and cytoplasmic fractions.

Supplementary data to this article can be found online at http://dx.doi.org/10.1016/j.bbamcr.2014.02.006.

## Figures and Tables

**Table 1:** The nuclear-to-cytoplasmic ratio is a more sensitive index than nuclear intensity to measure glucokinase translocation. Hepatocytes were incubated at either 5 mM or 25 mM glucose without or with 100 nM glucagon for 1 h and glucokinase localisation determined using immunostaining. Between 705 and 921 nuclei were imaged for each condition. Fluorescence (pixel intensity) is expressed as average ± s.d. with the coefficient of variation shown in parentheses. Changes between 25 mM glucose *vs*. 5 mM glucose and 25 mM glucose + glucagon *vs*. 25 mM glucose are expressed as percentage change. The cell number required to reach a statistically significant difference (p < 0.05) for the percentage changes shown was determined using sample size calculations (two independent samples, desired power = 0.90).

		Nuclear intensity	Cytoplasmic intensity	N/C ratio
Average ± s.d. (CV)	5glc	62.2 ± 27.0 (43)	7.9 ± 3.3 (42)	8.4 ± 3.3 (39)
	5glc + glucagon	57.3 ± 23.9 (42)	6.9 ± 2.6 (38)	8.8 ± 3.5 (40)
	25glc	43.8 ± 14.4 (33)	10.5 ± 3.7 (35)	4.4 ± 1.4 (35)
	25glc + glucagon	48.8 ± 17.7 (36)	9.1 ± 3.4 (37)	5.8 ± 2.3 (40)
Percentage change	25glc *vs*. 5glc	− 30	+ 33	− 48
	25glc + glucagon *vs*. 25glc	+ 11	− 13	+ 32
Cell number for p < 0.05	25glc *vs*. 5glc	47	37	15
	25glc + glucagon *vs*. 25glc	234	178	60
