# Supplementary figures and images for: Glucagon induces translocation of glucokinase from the cytoplasm to the nucleus of hepatocytes by transfer between 6-phosphofructo 2-kinase/fructose 2,6-bisphosphatase-2 and the glucokinase regulatory protein
Source: Biochim Biophys Acta. 2014 Jun;1843(6):1123–34. doi: 10.1016/j.bbamcr.2014.02.006 (PMC4024195; doi:10.1016/j.bbamcr.2014.02.006)

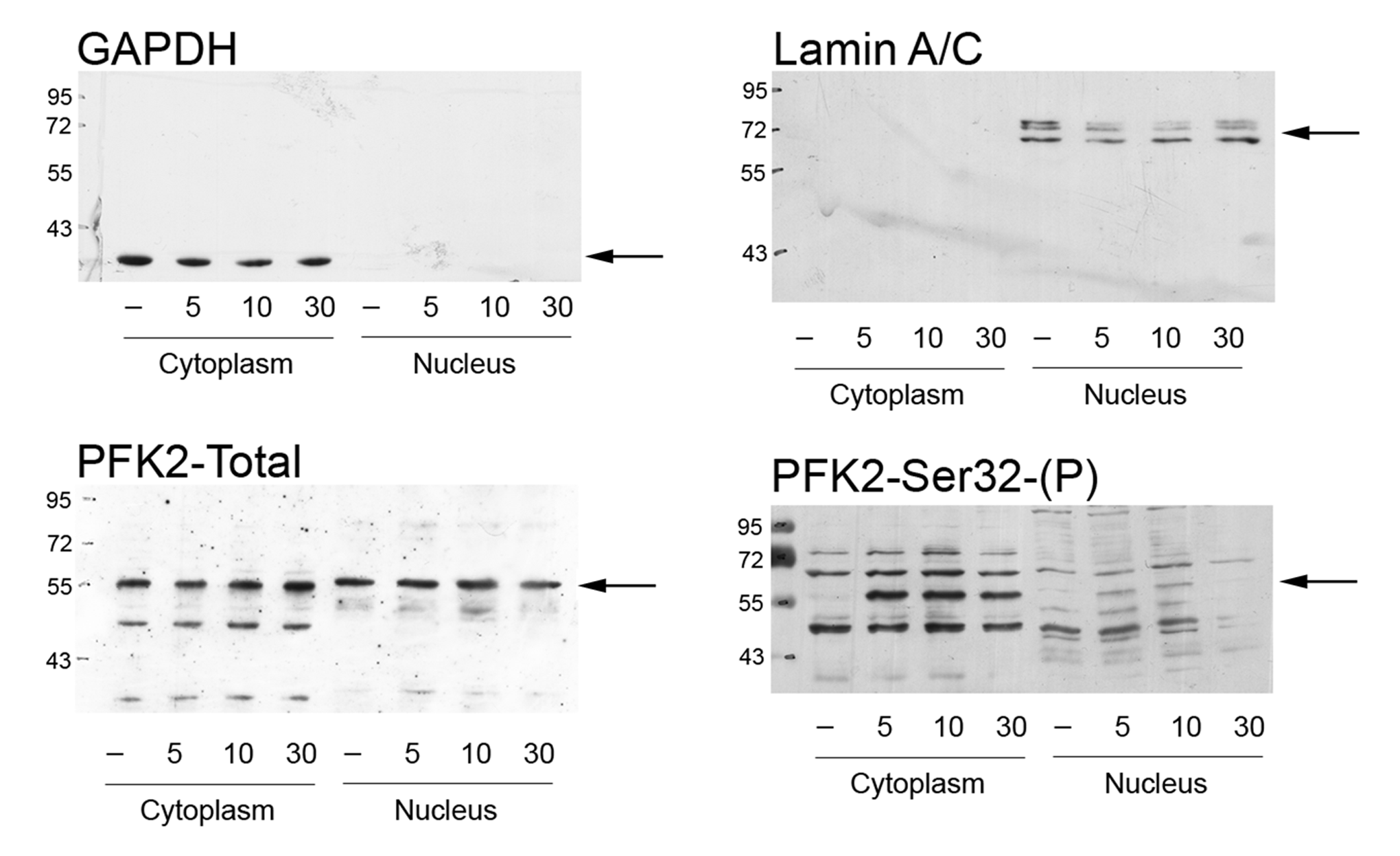

Supplement: Supplementary Fig. 1 — Localisation of PFK2/FBPase2 in hepatocytes. Hepatocytes were incubated with 25 mM glucose with or without 100 nM glucagon for 5, 10 or 30 min. The subcellular locations of GAPDH, Lamin A/C, total PFK2/FBPase2 and PFK2-Ser32(P) were determined by western blotting of nuclear and cytoplasmic fractions. [file mmc1.zip › bbr17201-mmc1.tif]
